# PROTOCOL: Bereavement Interventions for Children and Adolescents: An Evidence and Gap Map of Primary Studies and Systematic Reviews

**DOI:** 10.1002/cl2.70027

**Published:** 2025-03-22

**Authors:** Leonor Rodriguez, James Lyons, Amy Maloy, Ailsa O'Brien

**Affiliations:** ^1^ School of Health in Social Science University of Edinburgh Edinburgh UK

## Abstract

This is the protocol for a Campbell evidence and gap map. The objectives are as follows: (1) To identify and map all existing primary studies and systematic reviews (published and unpublished) on bereavement interventions/programmes for children and adolescents to create a live, searchable and publicly available EGM; (2) Provide a comprehensive descriptive overview of psychosocial outcomes targeted by bereavement interventions for children and adolescents; (3) Determine the characteristics of bereavement interventions targeted at children and adolescents, including age, location, duration, delivery, underlying theories, evaluation and target death.

## 1 Background

### 1.1 The Problem, Condition or Issue

Loss, grief and bereavement are terms often used interchangeably; however, they are conceptually different. Bereavement consists of having lost someone significant through death and grief consists of the psychobiological reaction to the loss, including the physical, cognitive and emotional responses that occur during bereavement (Shear et al. 2013; Ing et al. 2022). This loss may be associated with a person or a thing, not necessarily due to the death of a person and often coexists with a grieving process (Shear et al. 2013; Harris 2019).

The death of a loved one can be one of the most distressing and traumatic life events for children and adolescents (Melhem 2011; Alvis et al. 2023). Children and adolescents who experience a significant death understand the death in line with their age and stage of development (Speece and Brent 1984; Kaplow et al. 2010). Developmental staging of children's and adolescents' understanding of death does not explain how individual children and adolescents experience death; however, it is important to adopt a developmental approach when developing interventions targeted at children and adolescents (McClatchey and Wimmer 2018).

The number of children and adolescents experiencing a death from cancer is not known precisely; however, the Child Bereavement Network (Child Bereavement Network [CBN] 2024) estimates that 127 children are bereaved of a parent each day in the United Kingdom alone. According to Alvis et al. (2023), in 2015, the United Nations International Children's Emergency Fund estimated that globally, nearly 140 million children under the age of 18 had experienced the death of one or both parents. Considering other significant deaths, it has been estimated that 62% of children and adolescents will have experienced a bereavement by the age of 10 (Paul and Vaswani 2020). Therefore, bereavement in children and adolescents is a prevalent and important issue.

Bereavement can be a challenging experience for some children and adolescents. Contextual and environmental factors can impact children's and adolescents' reaction to grief. Some of these identified factors are the circumstances of the death, time since the loss, the relationship to the deceased, culture and ethnicity and exposure to prior life events (Santos et al. 2021; Harris 2019; Alvis et al. 2023). Research has identified that bereaved children and adolescents may present with eating and sleeping problems, enuresis, depression, anxiety, decreased school attendance, substance misuse, increased likelihood of self‐injury, separation anxiety disorder, conduct disorder and substance abuse (Fauth et al. 2009; Ridley and Frache 2020; Ing et al. 2022; Alvis et al. 2023; Woodward et al. 2023). The effects can be lifelong and can affect educational attainment and social development (Ridley and Frache 2020).

### 1.2 The Intervention

Due to the prevalence and significant and long‐lasting effects of bereavement on children and adolescents, effective bereavement interventions are needed. Preventative interventions are crucial, as these may reduce the risk of psychosocial issues associated with unresolved grief (Ing et al. 2022). There are examples of the diversity of interventions including web‐ (Zuelke et al. 2021), group‐ (Pfeffer et al. 2002), family‐based interventions (Sandler et al. 2013) and individualised interventions (Thienprayoon et al. 2015). The EGM is interested in all types of interventions as long as these are targeted at the bereaved child or adolescent. Heterogeneity is also found in who delivers the interventions, including mental health professionals, teachers, social workers, volunteers, among others (Thienprayoon et al. 2015; Zuelke et al. 2021); therefore, this information will also be captured.

Following previous systematic reviews in this area of research (Ing et al. 2022), this EGM is focused on identifying the impact of interventions on psychosocial outcomes in children and adolescents. Some of the outcomes of interventions identified in the literature are improved mood, reduced behavioural disorders, improved well‐being and better relationships with significant others (Yung‐Chi Chen and Panebianco 2018; Ing et al. 2022). There is, however, an inconsistency around the benefits of interventions across RCTs and non‐RCTs, due to measurement limitations, study designs and small sample sizes (Yung‐Chi Chen and Panebianco 2018; Ing et al. 2022). Benefits also vary according to gender, age, developmental stage, type of intervention, delivery method and the time between experiencing the bereavement and completing the intervention (Rosner et al. 2010; Duncan 2020). A systematic review focused on interventions available to adolescents and young adults bereaved by familial cancer only identified that interventions lack empirical evaluation using longitudinal and robust designs (Ing et al. 2022). Interventions also lack a clear underpinning theory to guide its development and application (Ing et al. 2022), for example, CBT and family systems theory.

### 1.3 Why Is It Important to Develop the Evidence and Gap Map (EGM)?

It is estimated that the majority of children and adolescents will adjust to bereavement without the need for an intervention; however, about 20% of them may benefit from effective interventions to prevent lasting poor outcomes (Currier et al. 2007). Adverse outcomes experienced by bereaved children and adolescents include poverty, poor social outcomes, reduced academic attainment and poorer mental and physical well‐being (Aguirre et al. 2024). The impact of a significant bereavement for children and adolescents can be very serious and long‐lasting. For example, Vaswani (2014) found that over 90% of young offenders in the prison system had experienced at least one bereavement, with two‐thirds of them having experienced over four. Bereavement interventions can be beneficial for children and young people (Ridley and Frache 2020), but these benefits can also reduce societal and financial costs associated with poor mental health and long‐term mental health disorders such as prolonged grief disorders (Alvis et al. 2023).

Bereavement interventions may be crucial in the lives of some children and adolescents who struggle with bereavement and grief. Despite this, there is a lack of a comprehensive and systematic tool that gathers the current existing evidence on interventions targeted at children and adolescents. This EGM will, therefore, provide a repository of the primary studies and systematic reviews on bereavement interventions for children and adolescents. The map will be created by using robust search, retrieval and methodological approaches to minimise potential sources of bias. It will be made publicly available and will provide a visual presentation of the existing evidence on bereavement interventions for children and adolescents.

The map will identify gaps in the evidence as well as highlight areas in which evidence is highly concentrated. This publicly available and visual resource can benefit (1) funders, who can assess the areas where the evidence is concentrated and identify the gaps in the knowledge, and target resources towards those areas; (2) practitioners and policymakers can access the map to see where evidence exists to inform policy and practice; (3) researchers can reduce research waste and duplication of research; and (4) members of the public can quickly access information that may be of relevance to them, for example, parents and families looking for suitable and evidence supports for their bereaved children and adolescents.

The EGM will comply with the standard EGM framework as a matrix. The rows will have the type of intervention/programme (e.g., group intervention, camp, individual psychotherapy, family therapy). The columns will contain the psychosocial outcomes identified (e.g., well‐being, improved mood, reduced stress, less depression, less anxiety). Additional information will be included in the map, which will enable to filter the map by mean age, region, delivery, type of death, underlying theory and intervention design. The elements specified in the framework will be coded into the EGM. These filters will be captured, as the benefits of interventions vary according to gender, age, developmental stage, type of intervention, delivery method and the time between experiencing the bereavement and completing the intervention (Rosner et al. 2010; Duncan 2020); therefore, it is important to capture this information in this EGM.

The EGM will also have a specific purpose, which is to inform the co‐creation of a mentoring, peer support intervention for adolescents who have experienced parental cancer. This EGM is part of the evidence that will support and inform adolescent stakeholders in the co‐creation of their intervention. As this EGM will help inform the co‐design of an intervention for adolescents, exploring the characteristics of existing interventions is important to provide adolescent co‐creators a clear understanding of the bereavement interventions landscape in areas such as outcomes, types, age, region, delivery, type of death, underlying theory and intervention design.

### 1.4 Existing EGMs and/or Relevant Systematic Reviews

To the authors' knowledge, there are no previous EGMs focused on interventions target at children and adolescents. There are several systematic reviews focused on different aspects of this topic. No limit to the period of the searches was determined a priori, as long as these were targeted at children and/or adolescents and published in English or Spanish (Table 1).

**Table 1:** Existing EGMs and systematic reviews.

Review	Outcomes reported	Comparison with this EGM
Yung‐Chi Chen and Panebianco (2018)	Behavioural and school problems, parent–child relationship, grief process, psychosocial functioning, externalising problems, hope, psychological distress, communicate feelings, grief expressions, psychological symptoms (anxiety, depression), other symptoms (immature regression, aggression, social withdrawal, irritability, school and learning problems), coping skills, emotions, relationships, cognitive symptoms.	Focused on children (3–5 years) and within a school context. This EGM is going to be targeting a more comprehensive age range and focus beyond school interventions.
Duncan (2020)	Open communication, Peer/social support, expressing emotion, role of adult (including relationships), conceptualising bereavement (meaning‐making), finding comfort, stress and trauma, looking to the future.	Focused on children only. Target stakeholders are teachers. This EGM is going to be targeting a more comprehensive age range and a wider range of stakeholders.
Ing et al. (2022)	Parent–child communication, coping strategy, expression of grief, mental health outcomes, psychosocial functioning, psychosocial well‐being, satisfaction in life, social support, social engagement, concentration. psychological (anxiety, depression, internalising and externalising problems, stress responses, prolonged grief and self‐esteem) and academic (achievement and performance), externalising problems (self‐reported, caregiver‐reported and teacher‐reported), decreasing internalising problems (teacher‐reported) and improving self‐esteem, grief‐related thoughts, depression, conduct disorder, social detachment, job aspirations, psychosocial functioning, traumatic grief, traumatic grief, PTSD.	Focused on familial cancer only, this EGM is more comprehensive. The age range is similar (15–25). This SR is limited by parent and sibling death; this EGM is more comprehensive.
Ridley and Frache (2020)	Psychosomatic and socioeconomic outcomes.	Focused solely on sibling death. This EGM will be more comprehensive in the types of death included.
Hanauer et al. (2024)	Grief, PTSD, depression, grief‐related stress. Grief education, coping techniques, peer support, family relationships, safe environment, comfort and healing, skills, future outlook.	Focused on children and adolescents but very focused on grief symptoms only. This EGM will be more comprehensive.
Rosner et al. (2010)	Grief, depression, anxiety, posttraumatic symptoms, social adjustment, well‐being, somatic symptoms.	Age range is children between 0 and 18 years. Focused only on quantitative outcomes, whereas this EGM will be more inclusive.
Lopez et al. (2017)	Grief treatment.	Focused solely on parental and sibling death, this EGM is more comprehensive. Design included must have a control group.

Several ongoing reviews were also identified. Arruda‐Colli et al. (2017) ‘Introducing communication about dying, death, and bereavement with children: a systematic review of children's literature’. PROSPERO 2016 CRD42016042129. Pereira et al. ‘Early interventions following the death of a parent: a mixed methods systematic review’. Wisker et al. ‘Facilitators and barriers of community‐based interventions for childhood bereavement: a systematic review and framework synthesis’. Finally, Pirl et al. ‘Systematic review of bereavement interventions for children whose parents died from cancer’. Due to the ongoing nature of these reviews, it was not possible to determine how these differ from the proposed EGM.

Overall, this EGM will therefore have a broader scope in terms of age and intervention types. It is important to notice that these systematic reviews have a heterogeneous set of outcomes that they are reporting on and this will have to be captured in this EGM.

## 2 Objectives

1. To identify and map all existing primary studies and systematic reviews (published and unpublished) on bereavement interventions/programmes for children and adolescents to create a live, searchable and publicly available EGM.
2. Provide a comprehensive descriptive overview of psychosocial outcomes targeted by bereavement interventions for children and adolescents.
3. Determine the characteristics of bereavement interventions targeted at children and adolescents, including age, location, duration, delivery, underlying theories, evaluation and target death.

## 3 Methods

EGMs are a tool to prioritise research needs and to support evidence‐informed practice and policy decisions. The Campbell Collaboration methodological guidelines for EGMs will be adhered to (White et al. 2020) and the project will be conducted according to the following six stages:

1. Scoping and development of the EGM framework. This entails determining the primary dimensions, row (interventions) and column (outcomes) headings.
2. Systematic and comprehensive searches. Several relevant databases will be searched using documented search strings. Published and grey literature will be included. The search strategy will be developed in conjunction with an expert subject librarian and piloted.
3. Screening for eligibility (i.e., title, then abstract, then full text). Results of the searchers will be double‐screened and reported using a PRISMA diagram.
4. Data extraction. Will be carried out in duplicate. Basic study characteristics, intervention categories, filters and data required for quality appraisal will be extracted.
5. High‐level quality appraisal. Data required for AMSTAR 2 (systematic reviews) and the Cochrane Risk of Bias will be used to evaluate the quality of randomised control studies.
6. Analysis according to the predefined inclusion/exclusion criteria. These dimensions are determined in the EGM framework. The rows will have the type of intervention/programme (e.g., group intervention, camp, individual psychotherapy, family therapy). The columns will contain the psychosocial outcomes identified (e.g., well‐being, improved mood, reduced stress, less depression, less anxiety). Additional information will be included in the map, which will enable to filter the map by age, duration, region, delivery, type of death, underlying theory and intervention design.

### 3.1 EGM: Definition and Purpose

EGMs are systematic evidence synthesis products that display the available evidence relevant to a specific research question (White et al. 2020). EGMs are used to identify gaps in the knowledge and responding to this gap by providing new evidence and studies for potential systematic reviewing, increasing discoverability and use of this existing material to inform decision‐makers, policy‐makers, commissioners and researchers to generate evidence‐based policy and guidelines (White et al. 2020).

This EGM is important, as it will provide a comprehensive description of the existing bereavement interventions for children and adolescents, which will help identify the characteristics of these interventions and identify outcomes that have been improved (or not) as a result. It has been found that the majority of children and adolescents will adjust to bereavement without the need for an intervention; however, about 20% of them may benefit from effective interventions to prevent lasting poor outcomes (Currier et al. 2007).

Bereavement interventions may be crucial in the lives of some children and adolescents who struggle with bereavement. Currently, there is a lack of a comprehensive and systematic tool that gathers the current existing evidence on interventions targeted at children and adolescents. This EGM will therefore provide a repository of the primary studies and systematic reviews on bereavement interventions for children and adolescents and identify gaps in the evidence as well as highlight areas in which evidence is highly concentrated.

This EGM will comply with the standard EGM framework as a matrix. The rows will have the type of intervention/programme (e.g., group intervention, camp, individual psychotherapy, family therapy). The columns will contain the psychosocial outcomes identified (e.g., well‐being, improved mood, reduced stress, less depression, less anxiety). Additional information will be included in the map, which will enable to filter the map by age, duration, region, delivery, type of death, underlying theory and intervention design.

This EGM will also have a specific purpose, which is to inform the co‐creation of a mentoring, peer support intervention for adolescents who have experienced parental cancer. This EGM is part of the evidence that will support and inform adolescent stakeholders in the co‐creation of their peer mentoring bereavement intervention.

### 3.2 Framework Development and Scope of the EGM

This EGM will comply with the standard EGM framework as a matrix. This EGM aims to be a descriptive but a comprehensive and visual representation of bereavement interventions for children and adolescents. The elements specified in the framework will be coded into the EGM. The rows will have the type of intervention/programme (e.g., group intervention, camp, individual psychotherapy, family therapy). The columns will contain the psychosocial outcomes identified (e.g., well‐being, improved mood, reduced stress, less depression, less anxiety). Additional information will be included in the map, which will enable to filter the map filter the map by age, duration, region, delivery, type of death, underlying theory and intervention design. The framework is a ‘living’ document and therefore new subcategories will be added during the data extraction process, based on the findings of the articles.

### 3.3 Stakeholder Engagement

This map is carried out as one of the components of a major study entitled: ‘Co‐Creation and Evaluation of the Bereavement Mentoring Programme for Adolescents (BMPA)’. This is a participatory study where adolescents will co‐create a bereavement intervention for their peers who experience parental death from cancer. This EGM, therefore, is part of the evidence that will support and inform stakeholders in the creation of their intervention. This EGM is underpinned by the aims and objectives of this study. However, Stakeholder engagement is not foreseen at this early stage; it will instead become part of the overall study, the scientific evidence available to support adolescents in the decision‐making and the creative process.

### 3.4 Conceptual Framework


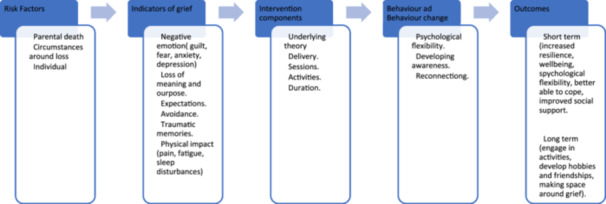


### 3.5 Dimensions

The inclusion and exclusion dimensions of this EGM are informed by the EGM framework. The key dimensions of this EGM are the type of intervention/programme (e.g., group intervention, camp, individual psychotherapy). Interventions must be targeted at children and adolescents specifically.

The psychosocial outcomes identified (e.g., well‐being, mood, stress, depression, anxiety, anger, coping, emotional distress, distress, expression of emotions, distress, grief symptoms, internalising problem, externalising problems, mental health, PTSD, resilience). Adverse psychosocial outcomes will also be coded (negative emotions, risky behaviours, sadness, stress), if reported in the sources. The specific dimensions are described in the dictionary of terms included in Appendix S2.

### 3.6 Types of Study Design

This EGM will include all relevant primary studies and systematic reviews (published and unpublished). Primary studies consist of individual studies where researchers generate and analyse their own data. Primary data can include both experimental (Empirical research involves an experiment in which data are collected in two or more conditions that are identical in all aspects but one [the manipulated variable] [Salkind 2012]) and non‐experimental designs (non‐experimental designs are those in which an experimenter describes a group or examines relationships between pre‐existing groups. The members of the groups are not randomly assigned and an independent variable is not manipulated. Therefore, no conclusions about causal relationships can be drawn [Salkind 2012]), which will be sought from several sources, including scientific journal articles, preprints, books, book chapters, reports and unpublished reports. Experimental designs may include independent measures, repeated measures, matched pairs and quasi‐experimental designs. Non‐experimental designs are usually observational or descriptive in nature. These may include correlational designs, cross‐sectional designs, observational research and qualitative research.

Systematic reviews, consisting of a review of primary studies adopting a systematic approach and screening with explicit inclusion, coding and reporting criteria, will also be included in this EGM.

### 3.7 Types of Interventions/Problems

This EGM will include interventions or programmes targeted at a child or adolescents who experienced the death of a significant other (parent sibling, other family member, friend) or pet. The target age is between 0 and 24 years of age, as defined by Patton et al. (2016).

Interventions can be of any kind; for example, these can be individual or group programmes, camps and individual psychotherapy, as this information about the types, duration, underlying theory and delivery of the interventions will be extracted as well.

Included interventions must be reported as a primary study. Primary studies are defined as an individual study where researchers generate and analyse their own data. These can have several types of study designs including, for example, quantitative methods, randomised‐controlled trials (random assignment to the intervention), case–control study, cohort study, cross sectional study, case reports and before versus after designs and series. Interventions can also have a qualitative design such as phenomenology, grounded theory, ethnography, historical, case study and mixed‐methods research.

Systematic reviews, consisting of a review of primary studies adopting a systematic approach and screening with explicit inclusion, coding and reporting criteria, will also be included. In addition to meta‐analysis and/or systematic reviews, rapid reviews and scoping reviews, both quantitative and qualitative, can be included.

Secondary data analyses, editorials, commentaries, opinion pieces, guidelines and policy documents on child and adolescent bereavement will be excluded. Any sources or articles not in English or Spanish will also be excluded.

### 3.8 Types of Populations

Children and adolescents who experience bereavement between 0 and 24 years of age. The top age range was defined by the definition provided by Patton et al. (2016). Any studies included in this age range can be included in the EGM. Age categories will be created to extract this information (0–9 children, 10–14 early adolescence, 15–19 late adolescence and 20–24 young adulthood).

### 3.9 Types of Outcome Measures

The purpose of this EGM is to provide a visual summary of the current landscape of outcomes targeted and measured in bereavement interventions for children and adolescents. Based on previous systematic reviews, it is important to acknowledge that there is a great variety in outcomes measured and associated with bereavement. For example, Yung‐Chi Chen and Panebianco (2018) and Ing et al. (2022) report on over 20 different outcomes.

This EGM will help adolescents involved in the design of a peer mentoring bereavement intervention, to select the outcomes that they believe are the essential ones based on their own lived experience. For this process to be successful, adolescents require a comprehensive tool (this EGM) that provides them with the full landscape of the outcomes to inform discussion and ensure that the new intervention is originated from a solid evidence base as well as lived experience.

The columns will contain the psychosocial outcomes identified (e.g., well‐being, mood, stress, depression, anxiety, anger, coping, emotional distress, distress, expression of emotions, distress, grief symptoms, internalising problems, externalising problems, mental health, PTSD, resilience). Adverse psychosocial outcomes will also be coded (negative emotions, risky behaviours, sadness, stress), if reported in the sources. The specific outcomes are in the dictionary of terms included in Appendix S2.

Additional information will be included in the map, which will enable to filter the map by age, duration, region, delivery, type of death, underlying theory and intervention design. As this EGM will help inform the co‐design of an intervention for adolescents, exploring the characteristics of existing interventions is important to provide adolescents co‐creators a clear understanding of the bereavement interventions landscape. It has also been identified that the benefits of interventions vary according to gender, age, developmental stage, type of intervention, delivery method and the time between experiencing the bereavement and completing the intervention (Rosner et al. 2010; Duncan 2020); therefore, it is important to capture this information in this EGM.

The framework (Appendix S2) is a ‘living’ document and therefore new subcategories will be added during the data extraction process, based on the findings of the included sources and articles.

### 3.10 Search Methods and Sources

To ensure the quality, reliability and applicability of this EGM, the literature retrieval methods will follow high‐quality standards. The systematic search will be designed by the research team and evaluated and informed by a subject specialist librarian. Considering the expertise of the team, only primary research and systematic reviews in English or Spanish will be included.

### 3.11 Electronic Databases

Electronic databases included are based on The University of Edinburgh database subscriptions, specifically focused on psychological topics. The databases are as follows:

- ASSIA (This database captures ProQuest, ERIC, Social Services Abstracts, ProQuest Dissertation and Theses Global).
- Ovid (This database captures APA, PscyINFO, Embase and Medline all).
- Web of Science Core Collection (This database captures the Social Sciences Citation Index and the Arts & Humanities Citation Index).
- Scopus (This database captures Medline and EMBASE).

Grey Literature:

- Overton
- Policy Commons
- Google Scholar

The search strategy will have to be adapted according to each of the listed databases. This will be carried out with the expert guidance of the subject specialist librarian. The proposed data search, specific for SCOPUS‐Web of Science interface, is included in Appendix S1.

### 3.12 Other Sources

The team will also search for grey literature across multiple sources. Grey literature consists of literature that is not published, not peer‐reviewed and generally harder to access. There are several sources of grey literature, for example, government reports, privately funded research, commissioned research, conference proceedings, working papers and dissertations.

Overton and Policy Commons will be searched for grey literature. Google Scholar is an important source of grey literature, including government reports and working papers. Searches in Google Scholar are restricted to 256 characters. Only the first 1000 records will be exported into the EGM, as this has been established as an acceptable number to capture the most relevant results (Miller et al. 2023). The search strategy suggested is as follows:

(bereavement)(child*¦adolescent*¦young person*)(intervention*¦ programme*)

The team will also search for relevant, systematic reviews and meta‐analyses via The Campbell Library, The Evidence for Policy and Practice Information and Co‐ordinating Centre (EPPI‐Centre) and The Social Care Institute for Excellence (SCIE). We will also search PROSPERO (University of York) for any protocols relevant to the EGM. Additionally, the team will hand search the reference lists of all relevant systematic reviews to identify any eligible studies.

### 3.13 Analysis and Presentation

#### 3.13.1 Report Structure

The output will be an EGM. It will have an accompanying report with tables and figures showing the types of interventions and psychosocial outcomes identified. The data will also be written based on the selected filters: age, region, delivery and so forth.

#### 3.13.2 Filters for Presentation

Filters: intervention characteristics (age, duration, region, delivery, type of death, underlying theory and intervention design) that can be applied to the map to show evidence relevant to those filters.

#### 3.13.3 Dependency

Each entry in the map will be a mapping study, mapping a specific domain of evidence. All publications (e.g., protocols and reports) that are part of the same study will only be included once on the map. Studies that cover multiple topic areas may appear multiple times within the map.

### 3.14 Data Collection and Analysis

The inclusion and exclusion criteria for this EGM are described in Table 2 below.

**Table 2:** Inclusion and exclusion criteria.

Inclusion	Exclusion
Interventions or programmes targeted at a child or adolescents who experienced the death of a significant other (parent sibling, other family member, friend) or pet. 0–24 years old. Any modality (e.g., individual or group programmes, camps, individual psychotherapy). Any duration. Any underlying theory. Any delivery mode (e.g., face to face or online). Study design primary studies (quantitative and qualitative) and systematic reviews (rapid reviews, ‐scoping reviews, meta‐ethnography). English or Spanish.	Secondary data analysis. Editorials. Commentaries. Opinion pieces. Guidelines. Policy documents. Not in English or Spanish.

#### 3.14.1 Screening and Study Selection

Database searchers will be equally divided between the four reviewers. Once all searches are completed, all identified sources and articles will be imported into EndNote. Data from all members of the team will be collated and all duplicates will be removed to avoid duplication effort in the subsequent stages of the EGM. The studies and sources will be transferred to EPPI reviewer software to enable data screening and extraction in duplicate. Reviewers will be paired up to complete the different stages, title and abstract, full text and data extraction. Any discrepancies will be resolved by a third reviewer (a member of a different pair).

A manual search of significant interventions (such as Camp Hope, Camp Magic and the Family Bereavement programme) will be carried out to ensure that these have been captured in the search. Additionally, we will search for the existing systematic reviews identified in this protocol. This will enable us to identify if the search terms are comprehensive and precise enough.

#### 3.14.2 Data Extraction and Management

The research team has developed a data extraction tool. The tool is included in Appendix S2.

This is a ‘live’ tool and will continue to be developed and populated during the data extraction process.

Title and abstract screening as well as full‐text data extraction will be carried out by four reviewers in duplicate using the application EPPI Reviewer Web. To ensure consistency and achieve high interrater reliability, a pilot study will be carried out with a sample of 10% of included studies. Any discrepancies will be resolved by a third reviewer.

Due to the nature of gap maps producing a vast amount of information, if multiple studies are reported in the same publication, each separate study will be represented in the map separately. EPPI reviewer software has the limitation that studies cannot be merged.

Equally, if there are multiple reports of a single study, we will treat these as a single study as it will be very difficult to identify them and link them together.

Once coding is completed, data need to be cleaned and checked for precision. A random sample (10%) of studies will be selected to systematically check that the coding has been correctly applied.

#### 3.14.3 Tools for Assessing the Risk of Bias/Study Quality of Included Reviews

The methodological rigour of the systematic reviews will be assessed in duplicate using AMSTAR‐2 (Shea et al. 2017). The methodological rigour of RCTs will be evaluated using the Cochrane Collaboration's tool (Higgins et al. 2011). Discrepancies that emerge in the process will be resolved by a third reviewer.

#### 3.14.4 Methods for Mapping

EPPI mapper will be used to create an interactive map. This map will be piloted before making the final version available to the public online. This map will summarise and organise all of the existing evidence. Results will be presented visually in a way as to identify where the evidence exists, where it is missing and where the gaps in the knowledge are found. The rows of the EGM will have the types of intervention/programmes (e.g., group interventions, camps, individual psychotherapy and family therapy). The columns will contain the psychosocial outcomes identified (e.g., well‐being, improved mood, reduced stress, less depression, less anxiety). If possible, these will be presented as short‐ and long‐term outcomes. Additional information will be included in the map, which will enable to filter the map by mean age, region, delivery, study type and type of loss experienced.

Additional information will be included in the map, which will enable to filter the map by mean age, region, delivery, study type and type of loss experienced.

The map will be accompanied by a descriptive report presenting the main findings of the map and the implications for future research and policy. A plain language summary will also be included to facilitate and enable the understanding of a wider group of stakeholders who may benefit from the findings of the EGM.

## Author Contributions

Content: Leonor Rodriguez, James Lyons, Amy Maloy and Ailsa O'Brien. EGM methods: Leonor Rodriguez. Statistical analysis: NA. Information retrieval: Leonor Rodriguez, James Lyons, Amy Maloy and Ailsa O'Brien.

## Conflicts of Interest

The authors declare no conflicts of interest.

## Preliminary Timeframe

Approximate date for submission of the EGM: January 2025.

## Plans for Updating the EGM

This EGM will be updated every 2 years.

## Supporting information

Supporting information.

Supporting information.
