# Supplementary material for: PROTOCOL: Bereavement Interventions for Children and Adolescents: An Evidence and Gap Map of Primary Studies and Systematic Reviews
Source: Campbell Syst Rev. 2025 Mar 22;21(2):e70027. doi: 10.1002/cl2.70027 (PMC11929544; doi:10.1002/cl2.70027)
Supplement: Supplementary file 1 — Supporting information. [file CL2-21-e70027-s001.docx]

**Appendix 1: EGM Search strings**

***Example for SCOPUS-Web of Science**

(

(Child* OR youth* OR boy* OR girl* OR “young people” OR teen* OR “young adult”* OR adolescen* OR “emerging adult*” OR “young person*” OR “juvenile*”)

**AND**

((“causal design” OR “cluster design” OR “comparison condition*” OR “comparison group” OR “control condition*” OR “control group*” OR “controlled trial” OR “cluster* trial*” OR random* OR rct* OR “clinical trial*” OR “systematic review*” or “meta-analys*” OR “process evaluation*” OR “program* evaluation*” OR “treatment condition*” OR “treatment effectiveness evaluation*” OR “treatment group*” or “ treatment outcome*”)

OR

(singl* or doubl* or trebl* or trip*) ADJ3 (blind* or mask*)

OR

(evaluat* or prospective*) ADJ3 (study or studies))

AND

(bereavement OR bereav* OR Grief OR griev* OR grief* OR loss* OR mourning OR mourn*)

AND

(Intervention* OR program* OR course* OR practice* OR treat* OR camp*)

)

**Appendix 2: Dictionary of Terms**

Filters: study characteristics (e.g., study design, country, subpopulation) which can be applied to the map to show evidence relevant to those filters.

Framework: defines dimensions of the map. Row and column heading and filters.

Dimensions: row (intervention categories) and column headings (outcome domains).

**Study Designs**

| Primary study | Individual study where researchers generate and analyse their own data. It is an empirical research study conducted by the authors. |
| --- | --- |
| Systematic Review | Review of primary studies adopting a systematic approach (at least 3 databases, screening with explicit inclusion criteria, coding and reporting of all relevant findings. |
| RCT | Random assignment to the intervention, including natural experiments. |
| Non experimental design with comparison group. | Non experimental studies with comparison group including regression-based designs. |
| Before versus after design. | Pre and post outcome measurement with no comparison group. |

Add as needed.

**Publication status**

| Completed | Study findings are available in a published report or paper (not only journal, NOT conference abstract or conference presentations). |
| --- | --- |
| Ongoing | Research is still in progress. There is no published paper or report of study findings. |

**Country**

Add country name in alphabetical order.

Add as needed

**Target Population***

| Children | 0-9 |
| --- | --- |
| Early adolescence | 10-14 |
| Late adolescence | 15-19 |
| Young adulthood | 20-24 |
| Families with children |  |
| Families with adolescents |  |
| Families with children and/or adolescents |  |

* Patton G., Sawywer S., Santelli J., Ross D., Afifi R., Allen N., et al. (2016) Our future: a Lancet commission on adolescent health and wellbeing. *The Lancet Commissions, 387* (10036), 2423-2478. <https://doi.org/101016/A0140-6736(16)00579-1>

**Programme or Intervention**

| Programme |
| --- |
| Intervention |
| Therapy |
| Camp |

Add as needed

**Underpinning Theoretical Foundation**

| Play Therapy | Play therapy provides opportunities for children/ adolescents to play out their emotions and concerns. |
| --- | --- |
| Art Therapy | Artistic processes are therapeutic. Awareness, expression of energy and feelings. |
| Grief Therapy | Addresses the four tasks of grief in children: 1)accept the death has happened 2)express feeling s around loss 3)adjust to an environment without the deceased 4)let go and invest in future relationships/ things. |
| Cognitive Behavioural Therapy | Based on behavioural therapy. Changing or revising habitual responses to stimuli. |
| Trauma Focused Therapy | Clients have experienced trauma. Components include: 1) psychoeducation, 2) parenting skills, 3) relaxation, 4) affective expression and modulation, 5) cognitive coping and processing, 6) trauma narrative and processing trauma, 7) mastery of trauma reminders, 8) child-parent sessions, 9) enhancement of personal safety and safety skills training. |
| Individual Psychological Therapy (Clinical Psychology) | Individual bereavement psychological therapy. |
| Family Therapy | Whole family is involved in the treatment of one family member. Grieving child/ adolescent is part of a system of interacting parts. |
| Group psychotherapy | Group activities. Groups may be supportive, psycho-educational, counselling or therapy oriented. |
| Bereavement camp | Formats may vary (weekend, weeklong, one-day camps). |
| Non reported |  |

*McClatchey I. & Wimmer J. (2018) Bereavement camps for children and adolescents. Planning, Curriculum and Evaluation. London: Routledge

Add as needed BUT make sure these are not sub-types or synonyms of other theories.

**Relationship to the deceased**

| Parental death |
| --- |
| Sibling death |
| Other family member death (grandparent, aunt, etc.) |
| General bereavement |

Add as needed

**Form of delivery**

| Online |
| --- |
| Face to Face |
| Manualised |
| Non manualised |

Duration

| One session |
| --- |
| One year |
| Depends on the individual |

Add as needed

**Comparison**

| No comparison |  |
| --- | --- |
| No treatment |  |
| Treatment as usual |  |
| Waiting list |  |
| Attention control | where participants receive some contact from researchers but both participants and researchers are aware that this is not an active intervention. |
| Placebo |  |
| Inactive comparison |  |

Add as needed

**Evaluation**

| Pre/ Baseline |  |
| --- | --- |
| Pre and Immediate Post |  |
| Immediate Post |  |
| 6 month follow up |  |
| 12 month follow up |  |
| +12 months follow up |  |

**Questionnaire/ Survey Used**

Add as needed

**Intervention main objective (purpose)**

| Identifying emotions |  |
| --- | --- |
| Self-awareness |  |
| Responsible decision making |  |
| Impulse control |  |
| Stress management |  |
| Increased social support |  |
| Improved communication |  |

Add as needed

**Intervention provided by**

| Counsellor/ Therapist |
| --- |
| Teacher |
| Volunteer (community) |

Add as needed

**Outcomes**

| Anger |
| --- |
| Avoidance |
| Anxiety |
| Communication |
| Competence |
| Complicated/ Prolonged Grief |
| Coping |
| Depression |
| Dysregulation |
| Emotional distress |
| Expression of emotions |
| Family functioning |
| Fear |
| Functional impairment |
| Global Distress |
| Grief symptoms |
| Guilt |
| Hope |
| Hyperactivity and attention |
| Intrusive thoughts |
| Internalising/ externalising problems |
| Isolation |
| Meaning Making |
| Mental health |
| Mood |
| Negative emotion |
| Positive emotion |
| Post-loss growth |
| Post-traumatic stress |
| Post-traumatic growth |
| Prosocial Behaviour |
| Protective Factors |
| Psychosocial functioning |
| Quality of life |
| Quality of relationships |
| Resilience |
| Risky behaviour |
| Sadness |
| School (Grade point average) |
| Self-esteem |
| Social support |
| Somatic symptoms |
| Stress |
| Therapeutic engagement |
| Traumatic Grief |
| Wellbeing |
