# Supplementary material for: PROTOCOL: Bereavement Interventions for Children and Adolescents: An Evidence and Gap Map of Primary Studies and Systematic Reviews
Source: Campbell Syst Rev. 2025 Mar 22;21(2):e70027. doi: 10.1002/cl2.70027 (PMC11929544; doi:10.1002/cl2.70027)
Supplement: Supplementary file 2 — Supporting information. [file CL2-21-e70027-s002.docx]

**CYP-24-03**

**Protocol Title:** Bereavement interventions for children and adolescents: An evidence and gap map of primary studies and systematic reviews

**Peer Reviewer 1:** The manuscript deals with an important topic and I have only a few minor suggestions to improve it. Here are my comments and suggestions:

Thank you very much for your insights. They have been very useful. We have addressed most comments except one but we have included an explanation of why.

| Comment |  |
| --- | --- |
| Page 2, “The intervention”: In this section, the authors give examples of the variety of bereavement interventions, but family-based interventions are not mentioned in this description. My suggestion is to add family-based intervention to the description. | Reference has been added now. |
| Page 3, “Existing EGMS and/or relevant systematic reviews”: There is no description of how the authors limited the time period of the literature search for review articles. Please add a description of the boundaries in time for the literature search. | We did not limit the time period of the literature search for review articles. Clarification added. |
| “Methods”: In the analysis of the included studies, it is important to consider the type of loss experienced by the children, whether it is the loss of a parent and caregiver, or the loss of a relative or friend on whom the child is not totally dependent. | Definitely important, was added now to the protocol. |
| A general comment is that it is important to be open to seeing the outcomes not only at an individual level for the child but also at an interpersonal level. When a parent dies, it affects the whole family, including parenting. Interventions aimed at the family and the relationship between the child and the remaining parent/caregiver can lead to improvements in, for example, family communication. | Definitely agree with this statement however the scope of the search could get extremely big for the EGM. This is the reason why family interventions are part of the exclusion criteria for this EGM. But we are very happy to carry out more EGMs and systematic reviews with this focus (we actually are doing). |

**Peer Reviewer 2:**

| Comment |  |
| --- | --- |
| Background:  It is tricky to sum all bereavements together in making the case that it's a public health problem - the consequences of some bereavements are more evident than others - death of a parent or caregiver - death of an uncle, child in the neighborhood, etc. How will they handle differences between amount of evidence of one area as compared to another? | Yes, completely agree with this. The exploratory nature of the EGM will help us understand where the majority of evidence is concentrated and possibly inform more focused systematic reviews in the future where we can explore the impact of these differences in detail.  This is similar to a comment from the first reviewer. We added a clarification that this information will be collected. |
| The Intervention:  They will include interventions at all levels – this sounds reasonable and appropriate. How will systematic reviews or qualitative studies or ethnographies be coded in terms of the level or quality of evidence? | This is a fair point.  We will collect data on the type of study, to determine the types of methods and design used in this field. The reason we are not judging the quality of every single type of study again, comes down to the scope of the EGM and realistically the resources available versus the required. Following the guidelines for EGMs, this is acceptable:  *Critical appraisal of included studies is recommended for Campbell EGMs but not mandatory. It is also possible to conduct critical appraisal of some studies, for example, systematic reviews, while not conducting this for other, for example, primary, studies* (White et al., 2020, p. 9)  Clarification added in Section 4.  We are going to carry out the risk of bias analysis for rcts and systematic reviews, as these can (and should) provide a high level of evidence. |
| Conceptual Framework:  Risk Factors – They include the person who died – parent, child, etc. as well as the cause or circumstances of death – sudden, violent, illness – that are not independent – e.g. violent death to parent and parent death from an illness. How will they handle this | The nature of an EGM is descriptive, by definition (White et al., 2020). We will therefore be able to display available evidence (frequency) and identify any gaps. Handling this information, as such, is outside the scope of this EGM and any EGM. It can inform, however the need for more research and systematic reviews focused on the impact of these differences in the future. |
| Indicators of grief – Should this be the next focus? A death may have multiple immediate effects such as depression, anxiety; family changes, economic changes… Are these all treated as indicators of grief? | These have been identified as possible consequences of bereavement, yes.  The EGM is focused on bereavement, not grief. Part of the motivation to carry it out is the need to clarify the usual confusion between terminology. |
| Intervention components – They include both modality of intervention (e.g. groups, camps, family) and Theory of the intervention which I see as quite different. Theory generally refers to the specified immediate targets of the intervention that are hypothesized to lead to later outcomes. | Yes, agreed, these are completely separate.  The end of Section 1.1 specifies that we are interested in the underpinning theory that guides the development and application of the intervention, we added some examples to help. |
| Behavior and Behavior Change – That seems appropriate – although it could be subdivided – maybe in separate places as short term and long term – which is now included under outcomes | Yes, agreed. However, our capacity to deliver this will depend on the type of studies and if we do find longitudinal designs or not.  We are interested in identifying if studies have followed ups and the duration of these, however we do not expect this design to be the norm, the exceptions instead. Explanation added on Section 4. |
| Dimensions:  The term "non experimental studies is overly broad - it could include everything from manuals, case descriptions, books or chapters or websites describing various programs - What if any are the inclusion criteria to ensure that this disseminates more scientifically supported programs? | We refer to the definition of non-experimental designs as a type of research design only:   - *Nonexperimental designs include research designs in which an experimenter simply either describes a group or examines relationships between preexisting groups. The members of the groups are not randomly assigned and an independent variable is not manipulated by the experimenter, thus, no conclusions about causal relationships between variables in the study can be drawn. Generally, little attempt is made to control for threats to internal validity in nonexperimental designs. Non-experimental designs are used simply to answer questions about groups or about whether group differences exist.(Salkind N.(2012) Encyclopedia of Research Design. USA: SAGE)* DOI:https://doi.org/10.4135/9781412961288*)*   We added a footnote for clarification. |
| Underpinning Theoretical Foundation:  These are types of interventions – they are not theories of how programs should affect outcomes. | We are aware that some of these terms can be controversial, however there are definitions of these concepts as theory that can be found in the literature, and this is why we have included them as such.  For example:  Art therapy theory integrates psychological theory principles on pathology and the role of the client and therapist with an understanding of the kinesthetic, sensory, and organizing principles of art-making and materials. (Rastogi et al. (2022) Foundations of Art Therapy. Elsevier, <https://doi.org/10.1016/B978-0-12-824308-4.00009-0>) |

**Peer Reviewer 3:** Please see comments on attached PDF

| Sections within the document need to be strengthened especially considering that it is a global review. | Yes, we are happy to do this. |
| --- | --- |
| Could we ask the author to explain the conceptual framework? | We are unsure of what additional explanation is required. This follows the style of recently published EGMs by Campbell (e.g., Miller et al. 2023) and it is also what the EGM guidelines requests: *The framework: The framework for an EGM defines the dimensions of the map: row and column headings and filters*.: (White et al., 2020). |
| Request the authors to clearly define the experimental a non-experimental design? If they plan to include before and after study designs and whether qualitative studies will be considered? | A definition for experimental and non-experimental designs have been added.  Examples of these types of studies have been added. |
| Can you please ask the authors to clearly define the intervention categories in this section? | More detail has been added. |
| AMSTAR 2 is designed for rating systematic reviews. Which tool will be used to asses the primary studies. | Same response as above:  We will collect data on the type of study, to determine the types of methods and design used in this field. The reason we are not judging the quality of every single type of study again, comes down to the scope of the EGM and realistically the resources available versus the required. Following the guidelines for EGMs, this is acceptable:  *Critical appraisal of included studies is recommended for Campbell EGMs but not mandatory. It is also possible to conduct critical appraisal of some studies, for example, systematic reviews, while not conducting this for other, for example, primary, studies* (White et al., 2020, p. 9)  Clarification added in Section 4.  We are going to carry out the risk of bias analysis for RCTs and systematic reviews, as these can (and should) provide a high level of evidence. |
| Please add this information into the main protocol. | Added to the main text now (Section 3.3.1). |
| Will this be a different intervention category? If so, please include in the main protocol section and also define the outcome categories in the main section. | This issue has been addressed earlier. No, it is not a different intervention category. |
| Is this an outcome? If so, please define all the categories. Is (if?) attitudes will not be captures, nor the negative effects of bereavement. | Yes, these are some of the outcomes we know from the literature review and pilot.  No, these are not attitudes but actual intervention outcomes. Negative outcomes will be included if relevant, we do not know this at protocol stage, but are certainly open to this. |

**Methods Editor:**

| **1. Background Information Section** |  |
| --- | --- |
| The background information for your study is clearly written for a general audience and is thorough. For a minor revision, please consider using APA formatting for all your in-text citations. You might consider moving the conceptual framework here and providing more detail about how the conceptual framework will inform the evidence and gap map. For example, it is not clear if indicators of grief or contents of the interventions will be part of the map and coded in the EGM, or how all the elements of the framework will or will not be represented in the map | We have revised APA in text citations. We are happy to fox any others we may have missed. We added a paragraph about the framework in this section. |
| **2. The Intervention Section** |  |
| The interventions included in the EGM are unclear. In this section, please consider clearly defining the eligible interventions and the outcomes of interest. For example, it is unclear if your map will cover any and all web-based interventions, group-based and individualized interventions or if there are any minimum requirements for the types of interventions of interest here (for example, interventions need to have a minimum amount of contact time). Additionally, you write, “*Some of the benefits of interventions identified in the literature are improved mood, reduced behavioral disorders, improved well-being and better relationships with significant others (Yung-Chi Chen & Panebianco, 2018; Ing et al., 2022).*” Are these all of the outcomes of interest? | Additional information was added to this section. |
| **3. Why is it important to develop the EGM?** |  |
| In this section, you need to consider who your stakeholders are and the purpose of the map. “*This EGM will therefore provide a repository of the primary studies and systematic reviews on bereavement interventions for children and adolescents,* “ but this doesn’t align with the purpose of the EGM, to outline the evidence for interventions that are relevant for bereavement, in order to highlight gaps in scholarship. If the adolescents from the BMPA study are your stakeholders, then make sure you add them to this section and provide a rationale. | We have added this information to the protocol. |
| **4. Existing EGMS and/or relevant systematic reviews** |  |
| The purpose of this section is to situate this EGM in the existing literature. However, in the protocol, there is little discussion of how the systematic reviews discussed relate to the proposed work. In addition, a table might clarify the interventions and outcomes included in this section and their relationship to the proposed EGM; it is currently difficult to separate the references from the discussion. | This was specified and converted into to a table. |
| **5. Objectives** |  |
| The statement in the objectives section is more about the methods rather than the research question guiding the review. The objectives should be more specific such as “What interventions have been studied in the literature aimed at adolescents and children for social, emotional and behavioral outcomes?” You might consider adding a more specific list of interventions and outcomes that are relevant to this EGM. | A set of objectives have been specified. |
| **6. Methods** |  |
| In this section you state, “ *EGMs are a tool to prioritize research needs and to support evidence ‐ informed practice and policy decisions. The Campbell Collaboration methodological guidelines for EGMs will be adhered to (White et al., 2020) and the project will be conducted according to six stages: (1) scoping and development of the EGM framework; (2) systematic and comprehensive searches; (3) screening for eligibility (i.e., title, then abstract, then full text); (4) data extraction; (5) high ‐level quality appraisal of systematic reviews; (6) and analysis (according to the predefined inclusion/exclusion criteria).*” For each of these six stages listed, you need to outline details about how you will adhere to the methodology and be as detailed as possible. | Details have been added to each of the six steps. |
| **7. Evidence and gap map: definition and purpose** |  |
| This section should discuss why it is important to do this EGM, not discuss the reasons why EGMs are important. | Details have been added, however this section is now very similar to the “Why is it important to develop this EGM? |
| **8. Framework development and scope** |  |
| As discussed above, it is unclear how the framework informs the EGM. It is not clear if the elements of the framework will all be coded into the EGM, for example. | This has been clarified in the text. |
| **9. Conceptual framework** |  |
| The conceptual framework would complement the background information section. Also note that there are no citations to the theory supporting this framework. As discussed earlier, it is also unclear how this framework will inform the evidence and gap map. What elements of this framework will be used in the EGM? | The conceptual framework has been added to the background information. All element of this framework will be included in the EGM. |
| **10. Dimensions** |  |
| The dimensions of the map, including the dictionary of terms, needs to be clearly outlined. As discussed above, more details are needed about the eligible interventions and the potential range of outcomes of interest. In addition, it is unclear what might be considered adverse outcomes in this literature. Also, if you provide this in the appendix, reference it here. | Outcomes have been added to the dictionary of terms and specified in the text. |
| **11. Types of study design** |  |
| The protocol reads, |  |
| “*This EGM will include all relevant primary studies and systematic reviews (published and unpublished). To capture these studies, both experimental and non-experimental studies will be sought from several sources including scientific journal articles, preprints, books, book chapters, reports and unpublished reports.*” The term non-experimental can include many kinds of studies and in the social science literature may include qualitative studies. The protocol should be more specific here in describing the types of eligible designs. From other sections of the protocol, it is clear that only experimental studies are included and these could be single-group pre-post designs. A list of the eligible designs is needed given the differences across disciplines in how study designs are defined. The protocol should also include a rationale for the study designs that are eligible. | This section has been described in more detail also based on the feedback of another reviewer (above). |
| **12. Types of intervention/problem** |  |
| This section should be about the types of interventions eligible for the EGM, not about study design. What are the characteristics of eligible interventions? How will you define what interventions are eligible when screening? The protocol indicates that you will search for meta-analyses and scoping reviews of the effects of interventions on bereavement for children and teenagers, but that you will limit this search to studies that have an existing protocol. However, systematic reviews and meta-analyses in the social sciences rarely have an existing protocol with the exception of Campbell Reviews. Requiring systematic reviews to include protocols will limit the amount of evidence you can collect. | The inclusion criteria has been described in more detail.  Protocol statement was removed. |
| **13. Types of population (as applicable)** |  |
| Please provide more detail about the decision to use the upper range of adolescents at age 24. Will studies that include only young adults still be included? | Yes.  More clarification added. |
| **14. Types of outcome measures (as applicable)** |  |
| The range of outcomes and adverse outcomes should be described in more detail. It would be helpful to characterize these outcomes in terms of domains and more specific measures. This section also implies that mean age, region and delivery will be potential filters, but no rationale is provided for why these characteristics are important aspects of the interventions for the EGM. | Outcomes have been specified and included in the dictionary.  A rationale for the outcomes and additional characteristics that will be extracted has been added. |
| **15. Other eligibility criteria** |  |
| Are there other eligibility criteria for included primary studies or systematic reviews that are not described in earlier sections? If so, please include here, or if not, please delete. | Deleted |
| **16. Search methods and sources** |  |
| The reference provided for the rationale of how to search Google is not included in the references (Miller et al., 2023). Also note that recent work by Gusenbauer and Haddaway (2020) discusses the challenges and limitations of using Google for searching. Please consult with a search specialist about best practice for searching gray literature and Google Scholar. Please provide some of your search terms and boolean phrases here and reference your complete search terms that are in your Appendix in this section. | Reference included.  We have consulted a topic librarian about out search strategy and databases.  We will consider the article going forward, however Google scholar is not intended as the principal search system, but part of a more comprehensive set of databases. |
| Gusenbauer, M., & Haddaway, N. R. (2020). Which academic search systems are suitable for systematic reviews or meta-analyses? Evaluating retrieval qualities of Google Scholar, PubMed, and 26 other resources. *Research Synthesis Methods*, *11*(2), 181–217.<https://doi.org/10.1002/jrsm.1378> | Very useful, thank you for the reference. |
| **17. Analysis and presentation: Filters for presentation** |  |
| In the section on filters for presentation, a different set of filters is discussed here than appears in the eligibility section. Here the examples provided include study design, country and subpopulation, but these are not discussed in the background section, and no rationale is provided for why these would be important filters. | Filters have been specified in this section.  Rationale has been specified in the outcomes section and the why it is important to develop this EGM sections. |
| **18. Data collection and analysis: Screening and study selection** |  |
| The description of the screening process is incomplete. In detail, please outline your inclusion and exclusion criteria and the screening process for titles and abstracts and full text-review. You should include a table or list for inclusion and exclusion criteria. It appears that the title and abstract screening will be conducted by a single reviewer. What checks are in place to ensure that the title and abstract screening phase remains unbiased? Typically, two independent screeners are used at the title and abstract phase. The protocol then states that after title and abstract screening, all included studies will be screened in duplicate, but no details are provided about who will conduct the screening or how conflicts will be resolved. Will the team use any features that assist coding in EPPI-Reviewer such as text mining/LLM? | A table has been added.  Every stage will be carried out in duplicate. This has been clarified. |
| **19. Data collection and analysis/Data extraction and management** |  |
| This section should describe the data extraction methods, not the screening of titles/abstracts and full texts. There is little information about the coding process such as whether all eligible studies will be double-coded, who will do the coding, how conflicts will be resolved, and what software will be used. | The missing details have been added. |
| **20. Tools for risk of bias** |  |
| It is unclear why only the systematic reviews are being assessed for study quality. What is the rationale for excluding critical appraisal of the primary studies? If systematic reviews are being assessed, then primary studies should also be coded for quality, particularly if the review intends to include both RCTs and single-group experimental studies. | This has been explained above. |
| **21. Methods for mapping** |  |
| To clarify, an EGM outlines the interventions and outcomes that have been studied through primary studies and systematic reviews. The direction of the findings are usually not included as implied by the statement on the top of page 9, “For example, …improved mood, reduced stress, less depression…). Meta-analysis should be conducted if the direction and magnitude of the findings is the goal of the review. In this EGM, the goal appears to outline where the evidence exists, not the direction or magnitude of the findings. | Same response as above:  We will collect data on the type of study, to determine the types of methods and design used in this field. The reason we are not judging the quality of every single type of study again, comes down to the scope of the EGM and realistically the resources available versus the required. Following the guidelines for EGMs, this is acceptable:  *Critical appraisal of included studies is recommended for Campbell EGMs but not mandatory. It is also possible to conduct critical appraisal of some studies, for example, systematic reviews, while not conducting this for other, for example, primary, studies* (White et al., 2020, p. 9)  Clarification added in Section 4.  We are going to carry out the risk of bias analysis for RCTs and systematic reviews, as these can (and should) provide a high level of evidence.  This is in line with previous Campbell EGM protocols such as Miller at el. (2023). |

| 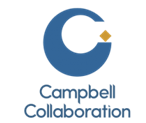 | Information Retrieval Methods GroupSystematic Review Protocol Peer Review Checklist |
| --- | --- |

Score

| **1. Will the appropriate subject databases be searched?** | **3** |
| --- | --- |
| Reviewer Comments:  Yes, the protocol states that they are going to conduct searches in a wide range of resources, including grey literature resources. | |

| **2. Will databases for related fields be consulted?** | **3** |
| --- | --- |
| Reviewer Comments:  I would suggest including clinical trial registers such as CENTRAL or clinical trials.gov | |

| **3. Did the authors consult the list of databases in the Campbell Searching for Studies Guide (Kugley et al., 2016)?** | **2/3** |
| --- | --- |
| Reviewer Comments:  In the methods section, the authors used a general reference to the Campbell guideline “The Campbell Collaboration methodological guidelines for EGMs will be adhered to (White et al., 2020)”  The reference is not included in the bibliography  Reference has been added. | |

## Grey Literature

| **4. Will the authors search for conference proceedings separately?** | **3** |
| --- | --- |
| Reviewer Comments: | |

| **5. Will the authors search for government documents separately?** | **3** |
| --- | --- |
| Reviewer Comments:  The authors commented that they will search for them via Google Scholar | |

| **6. Will the authors search for dissertations separately?** | **3** |
| --- | --- |
| Reviewer Comments:  ProQuest Dissertation and Theses Global | |

| **7. Did the authors consult the list of websites in the Campbell Searching for Studies Guide (Kugley et al., 2016)?** | 2 |
| --- | --- |
| Reviewer Comments:  The protcol didn´t specify if websites are going to be searched | |

## Supplementary Searches

| **8. Will the authors conduct a hand search of journals relevant to the topic?** | 1 |
| --- | --- |
| Reviewer Comments:  Hand searching is not contemplated in the protocol  We are going to carry a manual check to ensure significant interventions have been captured in the search. This has been clarified in the “screening and study selection” section. | |

| **9. Do the authors describe their plan for using web search engines (e.g. Google, Yahoo Search, Bing, Duck Duck Go, etc.) to locate web-based material?** | **3** |
| --- | --- |
| Reviewer Comments:  “Only the first 1000 records will be exported into the EGM, as this has been established as an acceptable number to capture the most relevant results (Miller et al., 2023). The search strategy suggests is as follows: (bereavement)(child*¦adolescent*¦young person*)(intervention*¦ programme*)” | |

| **10. Do the authors describe how they will use Google Scholar or other freely available scholarly search engines?** | **3** |
| --- | --- |
| Reviewer Comments: | |

| **11. Will the authors consult the reference lists of reviews and/or previous studies?** | **3** |
| --- | --- |
| Reviewer Comments:  “Additionally, the team will hand search the reference lists of all relevant systematic reviews to identify any eligible studies.” | |

| **12. Will the authors contact experts in the field?** | **1** |
| --- | --- |
| **12a. If so, do the authors provide a strategy for consulting experts?** |  |
| Reviewer Comments:  Due t o the exploratory nature of this EGM, hoping to inform the co-creation of an intervention by stakeholders with lived experience, we will not be consulting experts art this stage. | |

| **15. Will non-English studies be included?** | **3** |
| --- | --- |
| **15a.** : **If so, are non-English sources included in the search?** | **1** |
| **15b. If not, did the authors provide their rationale for not doing so?** | **1** |
| Reviewer Comments:  Spanish and English only, due to the knowledge of the reviewers. This was explained in the text. | |

# Search Strategy

## Bibliographic Databases

| **1. Is the full line-by-line search strategy listed for (at least) the main database?** | **3** |
| --- | --- |
| Reviewer Comments:  Yes, the search is provided for SCOPUS ( via web of sciences)  This database has not descriptors so they haven´t been used. It would have been better to develop the main search in a database with descriptors such as Medline | |

| **2. Do the authors state the platform in which the database will be searched?** | **1** |
| --- | --- |
| Reviewer Comments:  The authors added the platform only for some of the databases | |

| **3. Do the electronic searches appear sufficiently comprehensive for the topic?** | **1** |
| --- | --- |
| Reviewer Comments: | |

| **4. Does the search strategy use Boolean (AND/OR/NOT) and proximity operators appropriately?** | 3 |
| --- | --- |
| Reviewer Comments: | |

| **5. Does the search strategy include subject headings or descriptors appropriate for the topic**  **and the database?** | **1** |
| --- | --- |
| Reviewer Comments:  The main search was for Scopus conducted in WoS so they didn´t provide descriptors or subject headings  We consulted with a subject librarian. Descriptors or subjects were presented as alternative ways to carry out the search. We will use the piloted search terms that we have adapted to the different search engines. | |

| **6. Does the search strategy include keywords appropriate for the topic and the databases?** | 2 |
| --- | --- |
| **6b. Is truncation and phrasing (i.e. quotation marks) used appropriately?** | **3** |
| Reviewer Comments:  Some blocks of terms will need to add more terms or synonims | |

| **7. Is the syntax used appropriate for the database platform?** | 3 |
| --- | --- |
| Reviewer Comments: | |

| **8. Are line numbers combined appropriately?** | 3 |
| --- | --- |
| Reviewer Comments: | |

| **9. Are limits or filters used (such as date or publication type limits)?** | 1 |
| --- | --- |
| **9b. If so, is appropriate justification given for the use of limits and filters?** | **1** |
| Reviewer Comments:  The authors applied some filters to identify some of the study designs but those filters are not very sensitive and they do not include study designs such as qualitative studies or cohorts  If they are going to include a wide range of study designs perhaps is better do not apply any filter for study designs  The search terms have been piloted including and not including study design. Even with including study design this search terms we are at 63538. If we exclude designs this was an even larger number that was just impossible to process by a non-funded team. | |

# Other considerations

| **1. Was the information retrieval plan for this review sufficiently informed by the use of an information specialist?** | **3** |
| --- | --- |
| Reviewer Comments: | |

# General comments:

| Thanks for sending your protocol. Please, take into account the following considerations. The search has a risk of bias as it uses some terms to filter the study designs that do not include lots of study designs that could be object of this EGM:   - - - 1. Please, add the platform for each database.   These have been added.   - - - 1. PsycInfo, Embase and ERIC would need to be included. These databases appear with parentheses. I am not sure if that means that these databases perhaps are not going to be searched.   No, this means they are capture within this platform. So yes, ERIC, Embase and PsycInfo are included).   - - - 1. The search is using some kind of filter to identify different type of studies but that filter is not sensitive enough. If this EGM will include lots of study design such as systematic reviews, trials, observational studies and qualitative perhaps is better do not apply filters for study design. Perhaps the authors could consider adding a date limit for the searches. If you still would like to apply filters for each study design, please, check this website: <https://sites.google.com/a/york.ac.uk/issg-search-filters-resource/home>   This has been answered above.   - - - 1. Some blocks of concepts such as the populations and intervention blocks need to be improved. This block will need to include some psychotherapy that are well know such as CBT (cognitive behaviour therapy).   Not all programmes or interventions necessarily have such a specific underlying theory. We were cautious not to exclude interventions for this reason.   - - - 1. The population for this EGM is children, adolescents and young adults. Please, use the population in a consistent way through all the sections. For example, in the title the term “young adults” doesn´t appear.   This does not target young adults. Please refer to the definition we are using. We have specified it more clearly.   - - - 1. Please, check the content for the section dimensions. The information that is provided is about study designs but it would need to include information about the intervention/s.   We have discussed this in detail in other parts of the feedback provided earlier.   - - - 1. Please, check the bibliography. There are some references that do not appear.   Thank you, we have checked the references with the reference list. |
| --- |
|  |
